# A User‐Driven Framework for Dose Selection in Pregnancy: Proof of Concept for Sertraline

**DOI:** 10.1002/cpt.3429

**Published:** 2024-09-09

**Authors:** Charlotte Koldeweij, Caroline Dibbets, Bryony D. Franklin, Hubertina C. J. Scheepers, Saskia N. de Wildt

**Affiliations:** ^1^ Division of Pharmacology and Toxicology, Department of Pharmacy Radboud University Medical Center Nijmegen The Netherlands; ^2^ Department of Obstetrics and Gynaecology Maastricht University Medical Centre Maastricht The Netherlands; ^3^ Centre for Medication Safety and Service Quality Imperial College Healthcare NHS Trust London UK; ^4^ Department of Practice and Policy UCL School of Pharmacy London UK; ^5^ Grow, School for Oncology and Reproduction Maastricht The Netherlands; ^6^ Department of Pediatric and Neonatal Intensive Care Erasmus MC‐Sophia Children's Hospital Rotterdam The Netherlands

## Abstract

Despite growing knowledge of pregnancy‐induced changes in physiology that may alter maternal and fetal pharmacokinetics, evidence‐based antenatal doses are lacking for most drugs. Pharmacokinetic modeling and expanding clinical data in pregnancy may support antenatal doses. We aimed to develop and pilot a comprehensive and user‐driven Framework for Dose Selection in Pregnancy to support the clinical implementation of a best‐evidence antenatal dose for sertraline. After initial development and evaluation by experts, the framework prototype was piloted to formulate an antenatal dosing strategy for sertraline in depression and anxiety disorders. Next, the framework was reviewed and assessed for usability by a multidisciplinary working committee of end‐users comprising healthcare practitioners, experts from other disciplines including pharmacometrics, reproductive toxicology and medical ethics, alongside pregnant women and a partner. The resulting framework encompasses the following: rationale for drug selection, a comprehensive analysis of pharmacokinetic and dose‐related efficacy and safety data, and implementation aspects including feasibility and desirability of the recommended antenatal dose based on a structured maternal and fetal benefit–risk assessment. An antenatal dose recommendation for sertraline, as a case study, was formulated using this approach and endorsed for clinical use by the working committee. Future applications of the framework for other drugs can further demonstrate its suitability for developing best evidence, acceptable and clinically feasible antenatal doses.

**Study Highlights**

**WHAT IS THE CURRENT KNOWLEDGE ON THE TOPIC?**

Despite a growing understanding of pregnancy‐induced changes in physiology that may alter maternal and fetal drug efficacy and safety, evidence‐based antenatal dosing guidance is lacking for most drugs.

**WHAT QUESTION DID THIS STUDY ADDRESS?**

Can an effective method for establishing evidence‐based, acceptable and clinically feasible antenatal doses be developed through a user‐driven approach?

**WHAT DOES THE STUDY ADD TO OUR CURRENT KNOWLEDGE?**

We introduce a user‐driven Framework for Dose Selection in Pregnancy. This framework aims to support the implementation of best evidence and in some cases, model‐informed antenatal doses, also integrating feasibility and acceptability considerations. The applicability of this framework is illustrated through its pilot use for sertraline.

**HOW MIGHT THIS CHANGE CLINICAL PHARMA‐COLOGY OR TRANSLATIONAL SCIENCES?**

In the absence of tools for determining evidence‐based and clinically feasible antenatal doses, this framework for antenatal dose selection can help improve maternal and fetal therapies, addressing a crucial public health gap. The proposed antenatal sertraline dose has been endorsed and disseminated for clinical use in the Netherlands.

Pregnancy‐induced changes in maternal physiology and placental transfer may warrant medication dose alterations. Maternal changes affecting pharmacokinetics include an increased plasma volume, augmented renal filtration and altered activity of drug metabolizing enzymes.^1^, ^2^ These pharmacokinetic changes, along with potential alterations in pharmacodynamics, may affect drug safety and efficacy during pregnancy.^3^ However, despite the widespread use of medication by pregnant women,^4^, ^5^ most drugs are used off‐label in pregnancy, and specifically researched antenatal doses are generally lacking.^3^, ^6^ Ethical concerns and the lack of obligation for pharmaceutical companies to conduct clinical studies in pregnant women for drug licensing have resulted in limited evidence to support antenatal dosing.^7^ Consequently, HCPs typically prescribe doses intended for non‐pregnant adults, or, sometimes, reduce these doses due to concerns over fetal harm.^3^ Given altered pharmacokinetics and pharmacodynamics during pregnancy, such practices may result in suboptimal treatment or toxicity for mother and fetus.^8^

In this context, the emergence of pharmacokinetic modeling as an additional source of evidence for antenatal dosing appears promising. Alongside traditional pharmacokinetic studies, population‐based and physiologically based pharmacokinetic models can simulate maternal and fetal drug exposures throughout pregnancy without the need for extensive clinical data.^9^, ^10^ The feasibility of establishing antenatal doses drawing on pharmacokinetic modeling, alongside clinical and animal data, is currently investigated under project MADAM (Model‐Adjusted Doses for All Mothers).

Antenatal dose selection presents several challenges that warrant a systematic methodology. Firstly, given limited efficacy and safety data in pregnancy, an alternative dose finding method is to extrapolate doses from the general adult population taking pregnancy‐related changes in pharmacokinetics and pharmacodynamics into account. Data from pharmacokinetic modeling may support this process. This requires specific guidance for most guideline committees that may lack such expertise.^6^ Secondly, pregnancy‐induced changes in pharmacokinetics and pregnancy‐adjusted doses may affect the balance between the maternal and fetal benefits and risks compared to standard adult doses. This requires a careful reassessment of the benefit–risk balance. Nonetheless, a framework for investigating antenatal doses is currently missing. Therefore, we aimed to leverage the insights of experts and patients convened under project MADAM to develop and pilot a Framework for Dose Selection in Pregnancy (FDSP) for the antidepressant sertraline. This framework aims to support the analysis of pharmacological evidence alongside other considerations for implementing antenatal doses.

## MATERIALS AND METHODS

### Definitions

Antenatal doses were defined as optimized doses for medications indicated for maternal and/or fetal use, including both prescription and over‐the‐counter drugs, either initiated or continued during pregnancy.

### Sources

Alongside clinical expertise, FDSP design drew on two additional sources. It first expanded on a method for the benefit–risk assessment of off‐label pediatric drug use and dosing (BRAVO).^11^ This method was derived from the Problem, Objectives, Alternatives, Consequences, Trade‐offs, Uncertainty, Risk attitudes, and Linked decisions (PrOACT‐URL) benefit–risk appraisal methodology used by the European Medicines Agency to inform regulatory decisions on pharmacotherapy.^11^, ^12^, ^13^ Second, FDSP design incorporated insights from a stakeholder analysis among women and healthcare practitioners (HCPs), primarily from the Netherlands and Belgium. Drawing on focus groups,^14^ and online questionnaires^15^, this stakeholder analysis explored the acceptability and perceived barriers and facilitators for implementing model‐informed antenatal doses. It revealed a high willingness to use such doses among both groups if certain information needs were met. These included the rationales for altered antenatal doses and the quality of appraised evidence, including from pharmacokinetic models. Participating women had higher‐than‐average educational levels (43% with a university degree compared to 29% in the European Union), and 31% had a medical background.

### Iterative development

FDSP development occurred in three stages, following a user‐driven approach.^16^

#### Prototype development

The initial FDSP comprised four sections. Section 1 on “Drug selection” examined the rationale for investigating a dose in pregnancy. Section 2 on “Dose selection” aimed to compile and analyze all pharmacokinetic, efficacy and safety data related to antenatal dosing, alongside information on current drug administration and dosing practices. Section 3 on “Dose implementation” was designed to evaluate the acceptability of proposed doses considering maternal and fetal risks and benefits, along with dose certainty and feasibility. Section 4 outlined the final “Dose recommendation” for clinical use.

#### Expert evaluation

The prototype was then reviewed by the following experts: one obstetrician‐gynecologist, one pediatrician‐clinical pharmacologist, one toxicologist, two pharmacometricians, and one drug formulary expert. Key aspects under evaluation included the relevance, comprehensiveness and clarity of the framework. The prototype underwent iterative adaptations through rapid cycle design (**Supplementary materials** **S1** **and** **S2**).^17^

#### Piloting for sertraline, evaluation and usability assessment by end‐users

**Figure 1:**
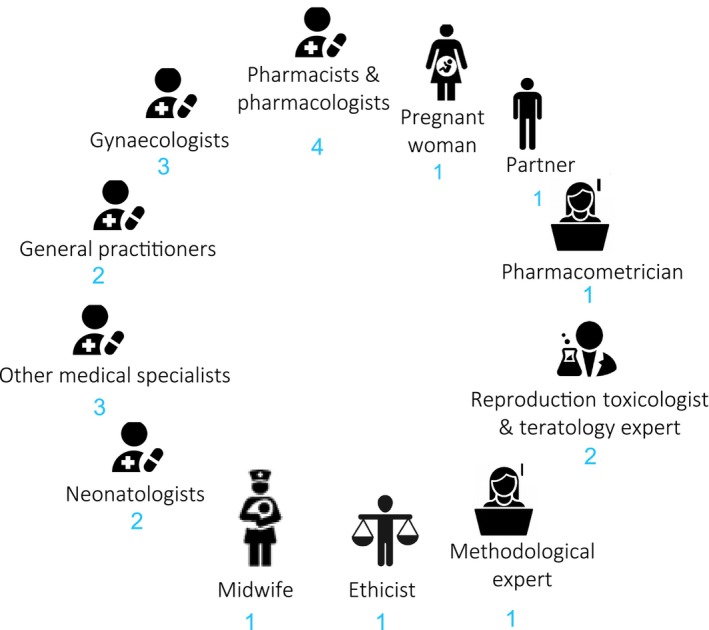
Composition of the FDSP and sertraline dose reviewing committee. FDSP, Framework for Dose Selection in Pregnancy.

Next, the FDSP was piloted for sertraline, chosen as a widely used antidepressant in pregnancy.^18^ Two researchers (CK, medical doctor and social scientist, and CD, medical student) supervised by HCJS, (obstetrician‐gynecologist) and SNW (obstetrician and pediatrician‐clinical pharmacologist), completed the framework for sertraline. Assembled under project MADAM, a national committee of 22 experts and representatives with diverse experience (**Figure** **1**), appraised the proposed sertraline dose for use in the Netherlands. This multidisciplinary committee offered both written and plenary feedback on the relevance, comprehensiveness and user‐friendliness of the FDSP applied for sertraline (**Supplementary material** **S1**). Framework usability was rated using the System Usability Scale, ranging from 1 (strongly disagree) to 5 (strongly agree).^19^

## RESULTS

### Generic framework

**Figure 2:**
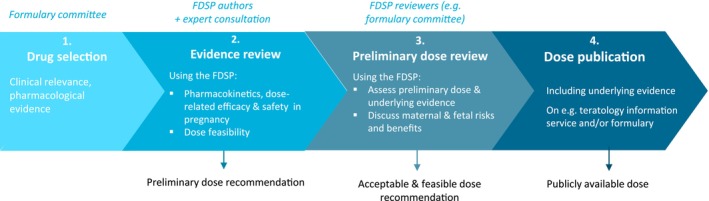
Steps for establishing an antenatal dose using the Framework for Dose Selection in Pregnancy. FDSP, Framework for Dose Selection in Pregnancy; MELINDA, Model‐Informed Dosing for All.

The resulting framework is presented in **Table** **1**. The antenatal dose review process using the FDSP is delineated in **Figure** **2**. The first stage involves selecting a drug for antenatal dose review. Drug selection criteria may include clinical factors such as frequency of use in pregnancy, evidence of suboptimal treatment with current doses, and availability of pregnancy‐related pharmacokinetic data. Framework completion follows a modular approach, allowing for questions to be selectively addressed depending on the drug indication, data availability and formulary needs. Data for framework completion can be obtained from a general literature search, supplemented by targeted searches and insights from clinical experts. Following appraisal by the reviewing committee, the completed FDSP should be amended to reflect input from reviewers before publishing the dose, if deemed ready for clinical use.

**Table 1:** Framework for dose selection in pregnancy

Questions	Information sources
**Part 1 – Proposed antenatal dose & questions to the formulary committee**	
1.1. Provide the title and hyperlink to the website page or other clinical resource on which the dose will be published.	e.g. Teratology information service
1.2. Outline the proposed dose.*	
*If a clinically usable dose recommendation cannot be issued, briefly outline remaining knowledge gaps and how to address them.	
1.3. Outline remaining questions for discussion by the formulary committee.*	
*Include obtained answers in the final version of the framework.	
**Part 2 – Drug selection**	
2.1. Which drug and indication(s) are under consideration?	Formulary committee
2.2. How often is the investigated drug used for the chosen indication(s) in pregnancy?	Epidemiological studies
2.3. Are there alternative interventions including drugs for the same indication(s) and how does the investigated drug compare to these alternatives for the chosen indication(s)?	Clinical guideline, clinical expert
2.4. Are there reasons to investigate the currently used dose in pregnancy based on the pharmacokinetics, mechanism of action and/or clinical experience?	Clinical pharmacologist, prescribing physician, brief literature review
**Part 3 – Dose selection**	
*3.1. Use and safety information in pregnancy*	
3.1.1. Is the drug registered for use in pregnancy for the chosen indication(s)?	SmPCs
3.1.2. What is the safety assessment of the drug in pregnancy?	Teratology information service
*3.2. Pharmacokinetics and dosing (general)*	
3.2.1. What are administration route(s) for the chosen indication(s)?	For 3.2.1–3.2.3 SMpCs, pharmacological databases, literature search, clinical guidelines, clinical experts
3.2.2. Which formulation(s) of the drug is(are) used for the chosen indication(s)? What are practical considerations for dosing?	
3.2.3. What is the recommended dose (range) for the chosen indication(s)?	
3.2.4.What is known about the absorption, distribution, metabolism, and elimination of the drug?	For 3.2.4–3.2.7 SmPCs, pharmacological databases, clinical pharmacologist, targeted literature search
3.2.5. Does the drug have an active metabolite? If so, describe its pharmacokinetic and pharmacodynamic properties.	
3.2.6. Is there a therapeutic range for the chosen indication(s) of the drug and how was it determined? In the absence of such a range, how was the dose determined and what is known about a dose‐exposure‐response relationship?	
3.2.7. Are there (patho)physiological factors of influence on the dose‐exposure‐response?	
3.2.8. How much interindividual variability is there in the dose‐exposure‐response?	For 3.2.8 and 3.2.11 clinical guideline, clinical pharmacologist, prescriber
3.2.9. Are there clinical or biochemical signs associated with underdosing or toxicity?	
3.2.10. Are laboratory analyses (e.g., TDM) routinely used to detect underdosing or toxicity?	TDM guideline
3.2.11. Is pharmacogenetic testing used for dosing the drug?	Pharmacogenetic guideline
3.2.12. What is the margin for intervention in the event of underdosing and toxicity?	
*3.3. Pharmacokinetics, pharmacodynamics, and dosing in pregnancy*	
*Pharmacokinetics*	
3.3.1. What are the expected effects of pregnancy‐induced changes in maternal physiology and metabolism, placental transfer, and fetal metabolism on the pharmacokinetics of the drug?	Reviews on pharmacokinetic changes in pregnancy, clinical pharmacologist
3.3.2. What can be learnt from pharmacokinetic studies of the drug in pregnancy regarding the aspects outlined in 3.3.1.?	Comprehensive literature search
*Pharmacodynamics*	
3.3.3. Is disease progression similar in pregnancy than in nonpregnant adults?	Clinical literature or expert
3.3.4. How does pregnancy influence the pharmacodynamics of the drug?	Targeted literature search, clinical pharmacologist
3.3.5. Summarize available data on the dose‐related efficacy of the drug in pregnancy.	For 3.3.5. and 3.3.6: comprehensive and targeted literature searches, clinical pharmacologist
3.3.6. Summarize available data on the dose‐related toxicity of the drug in pregnancy.	
3.3.7. Are there adverse events associated with use of the drug that may influence antenatal dosing?	Clinical and pharmacological databases
3.3.8. Are there drug interactions associated with use of the drug that may influence antenatal dosing?	Clinical and pharmacological databases
3.3.9. To what extent can postpartum changes in pharmacokinetics and pharmacodynamics inform the postpartum dose?	Targeted literature search, clinical pharmacologist
*3.4. Proposed dose*	
3.4.1. If a therapeutic range has been defined outside of pregnancy, can this range be used in pregnancy or do pregnancy‐related changes in disease progression, drug efficacy and safety warrant a pregnancy‐specific therapeutic range? In both cases, discuss whether a dosing strategy can be defined leading to appropriate maternal and fetal exposures.	
3.4.2. If no therapeutic range has been defined outside of pregnancy, can a dosing strategy be identified that matches observed exposures in non‐pregnant patients and that appears warranted based on available information on pregnancy‐related changes in disease progression, drug efficacy and safety?	
**Part 4 – Dose implementation**	
*4.1. Feasibility*	
4.1.1. How feasible is the proposed dose in the relevant setting(s) of care?	Clinical expert, FDSP reviewers
4.1.2. How is the proposed dose expected to affect current healthcare resources and processes?	Clinical expert, FDSP reviewers
*4.2. Desirability*	
4.2.1. What is the level of confidence in the proposed dose?	Data analyzed in part 3
4.2.2. What are the expected benefits of (not) implementing the dose?	Data analyzed in parts 2 and 3
4.2.3. What are the expected risks associated with (not) implementing the dose?	Data analyzed in parts 2 and 3
4.2.4. Are risk mitigation strategies available, feasible and warranted within the relevant setting(s) of implementation?	Clinical guidelines, clinical experts
4.2.5. What are the residual risks of implementing the dose and how do they compare to the residual risks of the current dose?	Clinical guidelines, clinical experts
4.2.6. Are there remaining knowledge gaps regarding the adequacy of the proposed dose?	Data analyzed in parts 2 and 3
4.2.7. Do the expected benefits, residual risks and the level of confidence in the proposed dose warrant its clinical use?	For 4.2.7 to 4.2.10: FDSP reviewers
4.2.8. Do the previous findings impact the ranking of the drug in the proposed dose compared to alternative interventions for the chosen indication(s)?	
4.2.9. Do the previous findings impact the selected route(s) of administration for the drug?	
4.2.10. What is the added value of the proposed dose for clinical practice?	
*4.3. Implementation*	
4.3.1. Is the proposed dose likely to be accepted by prescribers of the drug?	FDSP reviewers
4.3.2. Is the proposed dose likely to be accepted by pregnant women and their partners?	FDSP reviewers (patient representatives)
4.3.3. What are the next steps for implementation?	
Supplements	
1. Literature search string	
2. Quality & credibility assessment of pharmacokinetic simulations	
3. Version control	

FDSP, Framework for Dose Selection in Pregnancy; PBPK, physiologically based pharmacokinetic model; SmPC, summary of product characteristics; TDM, therapeutic drug monitoring.

### Framework piloting

The piloted FDSP for sertraline in the Netherlands is outlined in **Supplementary material** **S3**, as summarized below.

#### Part 1: Dose recommendation

The first FDSP section—previously section 4 in the prototype—outlines the final dose recommendation derived from subsequent analyses. The wording should convey the degree of certainty in available evidence following the Grading of Recommendations Assessment, Development, and Evaluation (GRADE) guidelines, as endorsed by FDSP reviewers.^20^

##### Antenatal sertraline dose

Patients should be informed of a possible decrease in the effect of sertraline during pregnancy, especially during the second and third trimester, due to increased sertraline metabolism. If symptoms worsen, a dose increase should be considered.

- For women who initiated sertraline use before pregnancy: maintain the pre‐pregnancy dose. If symptoms worsen during pregnancy, a dose increase should be considered, following titration steps for non‐pregnant adults. Doses exceeding 150 mg daily require careful consideration and discussion with the patient.
- For women starting sertraline during pregnancy: follow standard guidance for dose selection and titration. The antenatal dose recommendation for women who started sertraline before pregnancy otherwise applies.

Cytochrome P450 (CYP)2C19 variants can impact the required sertraline dose. Pharmacogenetic testing should be considered if unexplained side effects or an inadequate response to sertraline are reported during pregnancy, especially in women with a history of such issues for medications metabolized by CYP2C19:

- Normal or ultrarapid metabolizers: maintain the pre‐pregnancy dose in prior sertraline users, and adjust as needed based on response and side effects. For new sertraline users, follow guidance for non‐pregnant adults.
- Poor metabolizers: the same recommendations apply with a maximum dose of 150 mg daily.

Therapeutic drug monitoring is not routinely advised for sertraline in the Netherlands.

#### Part 2: Drug selection

This section specifies the indication(s) for which the dose is reviewed, alternative treatments and rationales for selecting the drug for antenatal dose review.

##### Drug and indications

Sertraline is a commonly prescribed antidepressant in pregnancy in the Netherlands and internationally.^18^, ^21^ Indications include depression and anxiety disorders.^22^, ^23^

##### Alternatives

Serotonin reuptake inhibitors (SSRIs) are a second‐line treatment for depression and anxiety disorders.^22^, ^23^ Initial or combined treatment involves non‐pharmacological interventions including psychotherapy. During pregnancy, the relative preference for non‐pharmacological interventions is greater to minimize potential fetal risks.^24^

##### Rationales for antenatal dose review

Pregnancy‐induced changes in sertraline distribution and metabolism may lower maternal exposure to the drug, potentially impacting treatment efficacy during pregnancy.^25^, ^26^

#### Part 3: Dose selection

This section comprises information regarding current use, safety and dosing of the drug along with pharmacokinetic and pharmacodynamic data relevant to antenatal dosing. Drug use aspects include administration route(s), standard doses and formulations for the chosen indication(s), and additional dosing considerations such as strategies to identify underdosing or toxicity. Pregnancy‐related information, including licensing details and available dosing guidance should be described, alongside maternal and fetal risks from antenatal drug use. Relevant pharmacokinetic data include the ADME (absorption, distribution, metabolism, and elimination) properties of the drug, maternal and fetal drug exposures throughout gestation, and placental transfer. Data can be extracted from traditional, physiologically based and population‐based pharmacokinetic studies. The quality of included pharmacokinetic studies can be graded using the adapted Jadad classification.^27^ Additionally, the FDSP comprises dedicated questions for appraising the credibility of population and physiologically based pharmacokinetic models for establishing model‐informed doses. Furthermore, information should be gained on a therapeutic range, or, in its absence, a routinely observed concentration range for the drug, alongside available data on dose‐related efficacy and safety. These concentration ranges may be described outside of pregnancy (in non‐pregnant adults or in neonates) and during gestation (in pregnant women and fetuses). Other relevant pharmacodynamic data encompass the influence of pregnancy on disease progression, and on target receptor expression and binding. Framework users should identify individual covariates that may influence antenatal dosing. Lastly, Part 3 includes details on pharmacokinetic and pharmacodynamic changes that may influence postpartum dosing. Taken together, these data should inform a dosing strategy that ensures adequate maternal and/or fetal exposure(s) throughout relevant gestational ages for the chosen indication(s). These exposures should align with the identified target concentration range(s), also considering pharmacodynamic changes in pregnancy. For drugs lacking a therapeutic range, appropriate antenatal doses may be extrapolated from standard adult doses by incorporating pharmacokinetic changes in pregnancy. For instance, a 30% reduction in maternal exposure may warrant a 30% dose increase compared to the standard dose assuming a similar concentration–response relationship. For fetal drugs, extrapolations regarding efficacy and/or safety could be made using neonatal data as a reference. The impact of such dose alterations on maternal and fetal drug safety is addressed in Part 4.

##### Use and safety in pregnancy

Sertraline is not licensed for use in pregnancy in Europe or the United States.^25^, ^28^ However, it is a preferred SSRI for pregnancy given a favorable safety profile.^29^ Neonatal adverse events associated with antenatal use of sertraline include persistent pulmonary hypertension of the newborn (PPHN) (baseline incidence 2 cases per 1,000 births; odds ratio 2.01 (95% CI 1.32–3.05) in sertraline‐exposed children after 20 weeks of gestational age) and moderate neonatal withdrawal symptoms, affecting 25 to 30% of neonates exposed to sertraline during the third trimester.^30^ While PPHN is a life‐threatening condition requiring multiple interventions,^31^ neonatal withdrawal symptoms are transient and self‐limiting. Due to these risks, it is recommended that fetuses exposed to sertraline in the third trimester be delivered at hospital.^29^ SSRI use may be associated with a slightly elevated risk of septal defects.^32^, ^33^ Study findings are inconsistent concerning a potential link with small‐for‐gestational‐age.^34^

##### Pharmacokinetics

In non‐pregnant adults, sertraline is administered orally at a 25–200 mg daily dose.^22^, ^28^ After initiating a low dose, sertraline dosing should be individually titrated based on a patient's response and potential side effects. Sertraline exhibits complex hepatic metabolism involving five CYP enzymes.^35^, ^36^ In non‐pregnant adults, interindividual variation in sertraline pharmacokinetics is most likely related to CYP2C19 polymorphisms, warranting CYP2C19 genotyping for specific indications.^37^ Fourteen pharmacokinetic studies in pregnancy (**Supplementary material** **S2**), and one maternal‐fetal physiologically based pharmacokinetic model were identified for sertraline.^38^ These studies, of heterogeneous quality, suggest that pregnancy‐induced alterations in maternal physiology may result in a 15–20% decrease in the maternal plasma concentration of sertraline, especially in the second and third trimesters, compared to baseline concentration. This decrease is most likely due to the combined effect of CYP enzyme induction (excluding CYP2C19) and a decreased albumin concentration during pregnancy.^26^, ^39^ Similar to non‐pregnant adults, maternal exposure to sertraline is marked by high interindividual variability likely derived from genetic variation in CYP2C19. Placental transfer of sertraline ranges between 30 and 40%.

##### Pharmacodynamics

Given large interindividual variation in dose–exposure and dose–response in non‐pregnant adults, a therapeutic range for sertraline is lacking.^40^ However, a dose–response relationship appears likely at the individual level. While data on dose‐related fetal toxicity are limited,^31^, ^33^ evidence suggests a dose–response relationship between moderate withdrawal symptoms and sertraline doses exceeding 100 mg in the third trimester (relative risk 3.31 (95% CI 1.05–10.39)).^41^ This dose–effect relationship may extend to other fetal or neonatal outcomes influenced by fetal development in the second or third trimester. This does not apply to septal defects, typically formed earlier in pregnancy.^42^

##### Conclusion

These pharmacokinetic findings imply that, to maintain sufficient maternal exposures, sertraline doses should not be reduced during pregnancy. If an inadequate or diminished clinical response is observed, particularly in the second or third trimester, sertraline dosing may need to be increased in certain women.

#### Part 4: Dose implementation

Lastly, this section examines the feasibility, desirability, and steps for implementation of the proposed dose. This entails appraising technical, logistical and ethical considerations to determine whether clinical implementation is feasible and warranted. Expert insights and perspectives from FDSP reviewers, including patients, should inform this assessment. First, dose feasibility considers factors such as drug formulation availability, preparation and administration, along with the resource implications of implementing the dose in relevant care settings. Next, the desirability of the dose can be assessed. First, this entails evaluating the level of confidence in this dose, as determined by the certainty of the reference concentration range used for dose selection, and an overall assessment of the relevance and quality of other underlying pharmacological data. Secondly, desirability of the proposed dose depends on the balance between established and plausible maternal and fetal risks and benefits. This benefit–risk assessment should consider available risk mitigation strategies, such as following up clinical symptoms of the treated condition, or therapeutic drug monitoring to detect underdosing or toxicity. Residual risks, which are the remaining risks when risk mitigation strategies are used, can then be identified.^11^ Benefits and residual risks should be weighed against each other, and against the benefits and risks of not implementing the proposed dose. Next, any remaining knowledge gaps regarding dose adequacy should be described. Drawing on this analysis, FDSP reviewers can assess whether the proposed dose can reasonably be implemented in clinical care. In instances that it cannot, practical recommendations on how to address the remaining knowledge gaps should be issued. Lastly, the acceptability of the proposed dose for HCPs and pregnant women should be considered and remaining implementation steps outlined.

##### Dose feasibility

The proposed antenatal sertraline dose falls within the standard dosing range for non‐pregnant adults.^25^, ^28^ It thus appears feasible across relevant care settings.

##### Dose desirability

Despite the moderate‐to‐low quality of available pharmacokinetic data, confidence in the proposed dose appears sufficient. Notwithstanding interindividual variation in sertraline pharmacokinetics and the lack of a well‐defined therapeutic range, available data suggest a likely decrease in maternal exposure during pregnancy for most women. Therefore, cautionary dose reductions, as sometimes practiced clinically,^43^ appear unwarranted. Instead, dose titration based on individual symptoms may be required in some cases, particularly in the second and third trimesters. Considering individual dose titration and the recommended antenatal dose falling within the standard dosing range for non‐pregnant adults (with 150 mg as a suggested maximum dose for potential CYP2C19 poor metabolizers), the likelihood of maternal overdosing appears low. Looking at fetal toxicity, the elevated risk of withdrawal symptoms, with a demonstrated dose–response relationship in the third trimester,^41^ should be balanced against the mild and self‐limiting nature of this condition.^44^ Although not proven, a dose–response relationship may exist for other neonatal outcomes, PPHN being most critical given its life‐threatening prognosis. Overall, given the self‐limiting course of neonatal withdrawal and the low absolute incidence and likely limited risk elevation of PPHN for increased sertraline doses within the standard range,^30^ these risks were deemed outweighed by the expected benefits of adequate maternal treatment through dose titration. Maternal benefits likely associated with higher sertraline doses comprise reduced risks of antenatal and postpartum depression. Indirect fetal and neonatal benefits include a reduced likelihood of perinatal complications linked to maternal stress, and enhanced postpartum bonding.^33^

##### Implementation steps

The endorsed sertraline dose has been published on the Dutch Teratology Information Service (TIS) and Dutch National Formulary.^45^, ^46^ Underlying evidence and considerations for dosing have been shared on a dedicated, openly accessible website.^47^ Dose adoption can be facilitated by educating clinicians and pregnant women about pregnancy‐induced changes in sertraline pharmacokinetics and the importance of maintaining adequate sertraline levels to keep depression and/or anxiety symptoms under control throughout pregnancy.

### Framework usability

Ten committee members, including HCPs, non‐clinical experts and patients, rated the usability of the FDSP. The willingness to use and integrity of the framework were rated high (median scores of 4.5 and 4.0 out of 5 respectively), with the framework scoring slightly lower on ease of use (median score of 3.5). The framework was deemed convenient to use (median score of 1.5 for “inconvenient to use”). Unnecessary complexity and the need for additional technical support received median scores of 2 and 2.5 respectively. Confidence in using the framework received a median score of 3.5, but committee members expected to learn how to use it quickly (median score of 4.0).

## DISCUSSION

We introduced a Framework for Dose Selection in Pregnancy, applied to sertraline and evaluated for relevance and usability by a multidisciplinary committee of experts and patients.

### Added value

This framework represents the first available tool to guide antenatal dose selection based on available evidence alongside dose feasibility and acceptability considerations. Tailored to facilitate dosing decisions under project MADAM, the FDSP accommodates usage by other formulary and guideline committees. As such, it may provide the impetus and necessary guidance for these committees to establish adequate antenatal doses, thereby helping to address an unmet health need.^3^, ^48^, ^49^, ^50^ While other dose selection frameworks exist focusing on pediatric dosing,^51^ or the general adult population,^52^ these are not fit for purpose for establishing best evidence, and potentially, model‐informed, antenatal doses. One reason is the need to balance risks and benefits of proposed doses for two individuals whose interests may not align, with limited evidence at hand.^7^, ^53^ Secondly, given scarce pharmacokinetic data in pregnancy,^48^, ^49^, ^54^ antenatal doses are likely to often draw on pharmacokinetic modeling and simulations.^9^, ^50^ However, despite recent attempts to introduce a blueprint for establishing model‐informed doses,^55^ comprehensive tools for appraising the credibility of pharmacokinetic simulations to inform clinical practice are lacking.^56^ To address these challenges, framework design drew on a stakeholder analysis on perceived barriers and facilitators for implementing model‐informed antenatal doses. Additionally, diverse stakeholders including HCPs, patients, and other experts participated to framework development, following a user‐driven approach. The obtained framework supports the assessment of various types of evidence, including from pharmacokinetic models. Drawing on clinical pharmacological principles,^19^ it can guide antenatal dose selection in the absence of a therapeutic range, as shown for sertraline. Beyond supporting a systematic evidence review, the FDSP outlines a structured and multidisciplinary approach for appraising the maternal and fetal risks and benefits associated with antenatal doses. It also provides a roadmap to implementation, as advised by others to increase adherence to clinical guidance.^57^ Lastly, it enables transparent reporting of dosing considerations to HCPs and patients, further promoting dose adoption.

While the pilot use of the FDSP for sertraline resulted in an antenatal dose within the standard adult dosing range, it provides an informative case for clinical practice. Previous research shows that without appropriate guidance, HCPs and women often reduce sertraline doses out of caution during pregnancy.^43^ Such practices partly stem from the lack of information on appropriate antenatal sertraline dosing. This analysis demonstrates that such dose reductions may result in inadequate treatment of maternal depression and/or anxiety symptoms, potentially harming the fetus indirectly. Based on available pharmacokinetic data, a dose increase of approximately 20% on average may appear warranted over the course of pregnancy. However, other factors were considered in recommending an adequate antenatal sertraline dose. These included the lack of a clear dose–exposure– response relationship, clinical experience that only some sertraline users undergo increased depression symptoms during pregnancy, and a weighted assessment of maternal and fetal benefits and risks associated with a raised sertraline dose during pregnancy. Considering these factors alongside pharmacokinetic evidence, we recommended increasing the sertraline dose only if a woman's depression symptoms deteriorate during pregnancy.

### Limitations

The proposed framework comes with several limitations. First, establishing antenatal dose recommendations following the proposed approach requires making assumptions when data is lacking, for example on a maintained exposure–response relationship in pregnancy. Clinical pharmacological expertise should be leveraged in those cases, and the benefit–risk balance, along with any remaining uncertainty regarding the proposed dosing strategy, should be transparently communicated to users. Second, framework completion appears time‐consuming given the extensive data analyses involved. While this was not undertaken for sertraline, the timeframe required for completion will be assessed in future FDSP applications. A simplified framework may be developed to enhance efficiency of use. Third, while we aimed to make the FDSP comprehensible for reviewers with diverse backgrounds, it may differ in accessibility for readers with varying clinical and pharmacological knowledge. However, the reviewing committee leveraged members' varied expertise to form an informed opinion collectively. This highlights the benefits of a multidisciplinary dose review also involving patients, to integrate diverse perspectives into complex decision‐making, as advocated by others.^58^ FDSP use by other formulary committees can offer more insights into the usability of this method. Fourth, while the FDSP was assessed for content validity by the MADAM working committee, further validation may involve establishing whether the resulting doses are associated with improved maternal and fetal outcomes compared to standard dosing. Lastly, the applicability of the FDSP for antenatal dose selection was assessed for only one drug, resulting in a dose recommendation that partly aligns with standard dosing in the general adult population. Available knowledge on pregnancy‐induced changes in pharmacokinetics suggests that future uses of the framework for other drugs may lead to antenatal doses that deviate more significantly from standard adult doses.^7^, ^8^, ^9^

### Implications for practice

Over the next 2 years, we aim to develop antenatal dosing guidelines for up to 20 drugs as part of project MADAM. This methodology will be integrated in the standard processes of the Dutch TIS to ensure the ongoing addition of new antenatal dose recommendations, and updates to existing ones when new data emerge. All the established doses and underlying data will be openly shared. Antenatal dose recommendations will be placed on the openly accessible websites of the Dutch TIS (https://www.lareb.nl/news/aangepaste‐doseringen‐tijdens‐de‐zwangerschap/) and Dutch National Formulary.^45^, ^46^ Additionally, they will be integrated into relevant clinical guidelines in the Netherlands.^59^ The underlying data will be shared on the newly created MELINDA‐dosing.com website (https://melinda‐dosing.com/).^47^ Established dose recommendations will be reviewed on a five‐yearly basis and in response to user feedback and input from working committee experts. Additional efforts to raise awareness and educate HCPs and pregnant women on the rationales and methods for establishing antenatal doses are ongoing.^60^, ^61^, ^62^, ^63^

By openly sharing this framework, we aim to support international guideline committees in establishing antenatal doses for a broader range of drugs. Although specific considerations for implementation may differ among countries, pharmacological analyses are likely to apply internationally. The FDSP invites its users to assess variability factors that may affect antenatal doses required in genetically diverse populations.^64^ Additionally, it can help identify implementation considerations across different settings.

## CONCLUSIONS

We presented a comprehensive and user‐driven framework integrating available evidence and implementation considerations to inform the selection of best‐evidence, acceptable, and clinically feasible antenatal doses, as exemplified for sertraline. Its application by other formulary committees and for other drugs may further illustrate its utility. In the absence of other tools for establishing adequate antenatal doses, this framework can help bridge an important public health gap faced by pregnant women and fetuses in accessing high‐quality pharmacotherapy.

## FUNDING

This publication is based on research funded by the Bill & Melinda Gates Foundation (INV‐023795). The findings and conclusions contained within are those of the authors and do not necessarily reflect positions or policies of the Bill & Melinda Gates Foundation. Part of this work was conducted during a work visit of CK to Imperial College Healthcare NHS Trust that was funded by a KNAW Van Leersum Grant/KNAW Medical Sciences Fund 2022, Royal Netherlands Academy of Arts & Sciences. BDF is funded by the National Institute for Health and Care Research (NIHR) North West London Patient Safety Research Collaboration. The views expressed are those of the author(s) and not necessarily those of the NIHR or the Department of Health and Social Care.

## CONFLICTS OF INTEREST

Dr. de Wildt receives compensation for consultancy work for Khondrion. All other authors declare no competing interests for this work.

## AUTHOR CONTRIBUTIONS

C.K., C.D., S.N.W. and B.D.F. designed the research. C.K. and C.D. performed the research. C.K., C.D., S.N.W. and H.C.J.S. analyzed the data. C.K., C.D., S.N.W., H.C.J.S. and B.D.F. wrote the manuscript.

## Ethics statement

None.

## Supporting information


Data S1, S2 and S3.
